# Adipose-Derived Stem Cells Pretreated With Phototherapy Promote HUVECs Migration and Angiogenesis by Mediating EYA1 Activation

**DOI:** 10.33549/physiolres.935620

**Published:** 2025-10-01

**Authors:** Dan ZHANG, Yi ZHANG, Jia WEN, Biling WU, Youwen CHEN, Yuejiao SONG, Chao LIANG

**Affiliations:** 1 Department of Anesthesiology, Zhongshan Hospital (Xiamen Branch), Fudan University, Xiamen, China; 2 Department of Anesthesiology, EYE & ENT Hospital of Fudan University, Fudan University, Shanghai, China; 3 Department of Anesthesiology, Zhongshan Hospital, Fudan University, Shanghai, China

**Keywords:** Phototherapy, Adipose-derived stem cells, Human umbilical vein endothelial cells, Angiogenesis, Migration, Eyes absent homolog 1

## Abstract

Promoting angiogenesis to enhance the success rate of parathyroid autotransplantation represents an effective strategy for improving patient outcomes following thyroid surgery. Eyes absent homolog 1 (EYA1) may be modulated by stromal vascular fraction (SVF) and adipose-derived stem cells (ADSCs) to facilitate angiogenesis. Phototherapy, which involves the use of artificial light source irradiation for disease prevention and treatment, has emerged as a promising approach. However, it remains unclear whether ADSCs pretreated with phototherapy can promote angiogenesis in the parathyroid gland through the regulation of EYA1. Primary human ADSCs (hADSCs) were isolated and identified. The impact of various wavelengths of light on the proliferation and secretion of angiogenic factors by hADSCs was assessed using a CCK-8 assay and an ELISA. Subsequently, the influence of light-pretreated hADSCs on HUVEC proliferation, migration, and angiogenesis was evaluated through CCK-8, Transwell, tube formation assays, and ELISA. Finally, qRT-PCR and Western blot analysis were employed to examine the effects of different wavelengths of light on the expression levels of differentiation-related transcription factors in hADSCs, including EYA1. To further elucidate the role of EYA1, an EYA1 interference plasmid (si-EYA1) and its negative control plasmid (si-NC) were transfected into hADSCs to determine whether silencing EYA1 would inhibit the promotion of HUVECs migration and angiogenesis by light-pretreated hADSCs. The results demonstrated that compared with green light (516 nm) and blue light (475 nm), red light (635 nm) irradiation significantly enhanced hADSCs proliferation and the secretion of angiogenic factors. Moreover, light-pretreated (red light) hADSCs markedly promoted HUVECs proliferation, migration, and angiogenesis. Additionally, red light irradiation significantly upregulated the mRNA and protein expression of EYA1, SIX1, TGF-β1, and Wnt1 while downregulating the mRNA and protein expression of DACH1 in hADSCs. However, silencing EYA1 attenuated the promotive effect of light-pretreated hADSCs on HUVECs migration and angiogenesis. These findings suggest that phototherapy-pretreated hADSCs may enhance HUVECs migration and angiogenesis *via* the activation of EYA1 and increased secretion of angiogenic factors.

## Introduction

Thyroid disease is a prevalent endocrine disorder with multifactorial etiology, including genetic predisposition, oncogene mutations, iodine deficiency or excess, and various lifestyle factors such as irregular diet, poor living habits, and psychological stress. With the increasing frequency of thyroid surgeries, the preservation of parathyroid function during these procedures has garnered significant attention [1]. During thyroidectomy, anatomical variations and surgeon inexperience can lead to inadvertent damage to the parathyroid blood supply or improper resection, resulting in inadequate preservation of parathyroid tissue and function. Consequently, patients may experience postoperative symptoms such as numbness, tingling, muscle spasms, and convulsions, which require lifelong management to maintain quality of life [2]. Although parathyroid hormone (PTH) replacement therapies are becoming available, their widespread use is limited by factors such as high cost, inability to accurately mimic physiological PTH levels, and potential risks like osteosarcoma [3,4]. Therefore, parathyroid autotransplantation has emerged as a critical preventive measure against permanent hypoparathyroidism following thyroidectomy. However, the low survival rate of transplanted tissue due to insufficient neovascularization remains a challenge [5]. Thus, investigating strategies to enhance angiogenesis and improve the success rate of parathyroid autotransplantation is an urgent scientific priority.

Adipose tissue comprises mature adipocytes and the stromal vascular fraction (SVF), which is rich in adipose-derived stem cells (ADSCs) with multi-lineage differentiation potential. ADSCs secrete a variety of growth factors and cytokines, offering broad prospects for applications in tissue engineering, organ repair, and medical cosmetics [6,7]. Early studies explored the induction of embryonic stem cells and palatine tonsil mesenchymal stem cells to obtain parathyroid cells capable of secreting PTH [8,9]. Subsequent research indicated that ADSCs possess the potential to differentiate into parathyroid cells, although no further related reports have emerged [10]. However, Cui *et al.* demonstrated that during parathyroid autotransplantation, the removal of periparathyroid adipose tissue reduced graft survival rates. In contrast, SVF and ADSCs enhanced the survival rate of parathyroid transplants by regulating the expression of eyes absent homolog 1 (EYA1) to promote angiogenesis both *in vitro* and *in vivo* [11,12]. The Eya1 gene, located on chromosome 8q13.3, is a highly conserved transcriptional coactivator that plays a critical role in both embryonic and neural development [13,14]. These findings suggest that ADSCs play a crucial role in parathyroid autotransplantation, with EYA1 being involved in the regulation of angiogenesis during this process.

Phototherapy has been extensively utilized in clinical settings. Beyond its role in disease prevention and promoting bodily rehabilitation, ADSCs combined with light therapy have demonstrated significant contributions to angiogenesis. For instance, Ginani *et al.* reported that low-intensity irradiation from an InGaAIP diode laser stimulated the proliferation of cryopreserved ADSCs [15]. Additionally, Park *et al.* found that low-level light irradiation could enhance the expression of various angiogenic factors, including VEGF, FGF, and HGF, in ADSC spheroids, thereby promoting ischemic wound healing[16]. However, it remains unclear whether phototherapy-preconditioned ADSCs can promote angiogenesis in parathyroid autografts and if this effect is mediated by the regulation of EYA1. Therefore, in this study, ADSCs were subjected to pretreatment with varying wavelengths of light prior to co-culture with human umbilical vein endothelial cells (HUVECs). This was done to investigate the effects of photopretreated ADSCs on HUVECs migration, angiogenesis, and EYA1 expression, as well as the underlying mechanisms. The objective is to provide a theoretical and practical foundation for further research into parathyroid autotransplantation.

## Methods

### Obtain and culture of cells

Adipose tissue was procured from two healthy female donors aged between 18 and 30 years, who provided written informed consent prior to undergoing abdominal liposuction for bariatric surgery. The adipose tissue was rinsed with phosphate-buffered saline (PBS) and subsequently digested with 0.1 % Type I collagenase (Aladdin, China) for 50 min as detailed in the literature [17]. Following digestion, the tissue was centrifuged to isolate the SVF. The SVF was then cultured in phenol red-free Dulbecco’s Modified Eagle Medium (DMEM, Thermo Fisher, USA) supplemented with 10 % fetal bovine serum (FBS) and 1 % penicillin/streptomycin (P/S), with media changes occurring every 48 h. Human adipose-derived mesenchymal stem cells (hADSCs) were successfully obtained after 3–4 passages of culture. This study was approved by the Medical Ethics Committee of Zhongshan Hospital (Xiamen Branch), Fudan University (Approval No. B2025-032).

HUVECs (Wuhan Pricella Biotechnology Co.,Ltd, China) were cultured in a humidified cell incubator (RWD, China) at 37 °C in an atmosphere of 5 % CO_2_. The culture medium consisted of phenol red-free Dulbecco’s Modified Eagle Medium supplemented with 10 % fetal bovine serum (FBS) and 1 % penicillin-streptomycin (P/S), and it was refreshed every two days.

### Flow cytometry

Flow cytometry was employed to characterize the surface markers of hADSCs. Specifically, hADSCs from the fifth passage were trypsinized and subsequently washed with PBS to prepare a cell suspension at a concentration of 1×10^9^ cells/l. The cell suspension was then incubated with fluorescein isothiocyanate (FITC) or phycoertyhrin (PE)-conjugated monoclonal antibodies against CD44, CD90, CD105, CD34, and CD45 (mouse anti-human, Abcam, UK), with each antibody added separately. A medium devoid of cells served as the negative control. Following a 20-minute incubation in the dark, the positive expression rates of the respective surface markers were quantified using flow cytometry.

### Adipogenic and osteogenic differentiation

hADSCs at the fourth passage were enzymatically dissociated using trypsin, and the cell concentration was adjusted to 2×10^5^ cells/ml. For osteogenic differentiation, the cells were cultured in an alkaline medium (Cyagen Biosciences Inc., China) supplemented with 1×10^−8^ mol/l dexamethasone, 1×10^−2^ mol/l β-glycerophosphate sodium, and 1.5×10^−4^ mol/l ascorbic acid for a period of two weeks. Osteogenic differentiation was confirmed by alizarin red staining. For adipogenic differentiation, cells were initially induced with medium A (Cyagen Biosciences Inc., China), containing 1 μmol/l dexamethasone, 10 mg/l insulin, 0.5 mmol/l 3-isobutyl-1-methylxanthine, and 100 mg/l indomethacin for three days. Subsequently, cells were induced with medium B, containing 10 mg/l insulin, for one day. This induction cycle with media A and B was repeated four times. Adipogenic differentiation was confirmed by Oil Red O staining.

### Phototherapy stimulates cells

hADSCs were seeded into well plates according to the specific requirements of each experiment. After the cells reached confluence, the wavelengths of the phototherapy LED lights (Repuls Lichtmedizintechnik GmbH, Germany) were set to 475 nm (blue light, BL), 516 nm (green light, GL), and 635 nm (red light, RL). The average irradiance for each light source was 40 mW/cm^2^, with a daily irradiation dose of 24 J/cm^2^ delivered over a 10-minute period. This treatment was administered once daily for a total of three consecutive days [18].

### Plasmid transfection

The siRNA sequence targeting EYA1 was designed (Table 1), and the two complementary oligonucleotide strands were synthesized based on this sequence. These strands were then cloned into a vector to generate the plasmid (HANBIO, China). The Lipofectamine 3000 transfection reagent (Thermo Fisher Scientific, USA) was utilized to introduce the plasmids si-EYA1 1#, si-EYA1 2#, and their corresponding negative control plasmid (si-NC) into hADSCs for transient transfection over a period of 48 h. Transfection efficiency was assessed using real-time fluorescence quantitative PCR (qRT-PCR) and Western blot analysis. The si-EYA1 plasmid exhibiting higher transfection efficiency was selected for further experimentation.

### Methods of cell intervention

To evaluate the effect of light pretreatment on the capacity of hADSCs to regulate HUVECs migration and angiogenesis, a Transwell co-culture system was established. HUVECs were seeded at a density of 8×10^4^ cells/well in the lower chamber of a 24-well plate and cultured until fully adherent. hADSCs were then seeded into Transwell chambers at a density of 1×10^4^ cells/well, with or without prior light pretreatment. The treated chambers were subsequently inserted into the lower chambers containing HUVECs, and differences in HUVEC migration and angiogenesis were analyzed after 24 h of co-culture. Subsequently, si-EYA1 and si-NC plasmids were transfected into hADSCs, followed by a 24-hour co-culture with HUVECs to investigate whether EYA1 silencing influenced the promotion of red light-pre-treated hADSCs on HUVECs migration and angiogenesis. Additionally, untreated HUVECs served as the blank group, while untreated hADSCs served as the control group.

### Cell counting kit-8 (CCK-8)

The cells were incubated until they became adherent and then grouped and treated as previously described. Subsequently, 10 μl of CCK-8 solution (Solarbio, China) was added to each well and incubated for 2 h. The absorbance at 450 nm was measured using a Varioskan LUX microplate reader (Thermo Fisher, USA). The proliferative activity of each group was calculated according to the specified formula.

### Enzyme-linked immunosorbent assay (ELISA)

hADSCs from each group were collected by trypsin digestion and subjected to ultrasonic disruption for cell lysis. The resulting lysate was centrifuged, and the supernatant was used for ELISA analysis. Following the manufacturer’s instructions (Elabscience, China), the supernatant from each group was added to a 96-well plate pre-coated with antibodies. Various working solutions were sequentially added according to the protocol. After terminating the reaction, the optical density (OD) values of each well were measured at a wavelength of 450 nm using a microplate reader. The concentrations of vascular endothelial growth factor (VEGF), basic fibroblast growth factor (bFGF), platelet-derived growth factor (PDGF), and transforming growth factor-β1 (TGF-β1) in the supernatant were determined using standard curves.

### Transwell assay

HUVEC suspensions from each group were prepared at a concentration of 1×10^5^ cells/ml. Following centrifugation, the supernatant was discarded, and 1 ml of serum-free medium was added to the pellet, which was then aliquoted into the upper chambers of Transwell (Corning, USA). These inserts were subsequently placed into the lower chambers of 24-well plates containing 10 % fetal bovine serum (FBS) complete medium and incubated for 48 h in a humidified incubator at 37 °C with 5 % CO_2_. After incubation, the cells that had migrated to the underside of the membrane were fixed with paraformaldehyde for 20 min and stained with 0.1 % crystal violet at room temperature for 10 min. Finally, the number of transmembrane cells per well was quantified by observing three random fields under an inverted microscope (OLYMPUS, Japan).

### Tube formation assay

Fifty microliters of diluted Matrigel (Corning, USA) was added to each well of a 96-well plate and incubated at 37 °C for 30 min to allow gel formation. Subsequently, cells from each group were suspended at a density of 2×10^5^ cells/ml and seeded onto the pre-coated Matrigel in the 96-well plates at a rate of 1×10^4^ cells per well. After an additional incubation period of 4 h, the number of tubular branch nodes in three randomly selected fields per well was quantified under a microscope. The average values were calculated to assess the angiogenic potential of the cells.

### qRT-PCR

Total RNA was extracted from each experimental group using Trizol reagent (Beyotime, China). The extracted RNA was reverse-transcribed into cDNA utilizing the BeyoFast™ SYBR Green One-Step qRT-PCR Kit (Beyotime, China). The resulting cDNA was then combined with gene-specific primers (Table 2) and nuclease-free water. Quantitative real-time PCR amplification was conducted on a QuantStudio^TM^ 5 real-time PCR system (Thermo Fisher Scientific, USA), following the manufacturer’s protocol. Relative gene expression levels were quantified using the 2^−ΔΔCt^ method.

### Western blot

Total proteins from each experimental group were extracted using RIPA lysis buffer (Beyotime, China) and quantified *via* BCA assay (Elabscience, China). Protein samples were denatured by incubation in a metal bath at 95 °C. Subsequently, 40 μg of protein was separated by electrophoresis on a 10 % SDS-PAGE gel and transferred onto a PVDF membrane. The membrane was blocked with 5 % skim milk powder for 1 h at room temperature. Primary antibodies against EYA1 (mouse polyclonal), DACH1 (rabbit polyclonal), SIX1 (mouse monoclonal), TGF-β1 (rabbit monoclonal), Wnt1 (rabbit polyclonal), and GAPDH (rabbit polyclonal) (all diluted 1:1000, Abcam, UK) were applied overnight at 4 °C. After washing three times with TBST, the membranes were incubated with horseradish peroxidase-conjugated goat anti-rabbit or anti-mouse secondary antibodies (1:5000, Abcam, UK) for 1 h at room temperature. Protein bands were visualized using ECL reagent and imaged using the GelDoc GO system (Bio-Rad, USA). The relative expression levels of target proteins were normalized to GAPDH as an internal control.

### Statistical methods

Data analysis and image generation were conducted using GraphPad Prism 8 software. Each experiment was independently replicated three times, with the results presented as mean ± standard deviation (mean ± SD). For comparisons among multiple groups, one-way analysis of variance (ANOVA) was employed, followed by *post hoc* LSD-*t*-tests for pairwise comparisons between groups. Statistical significance was set at *P*<0.05.

## Results

### Identification of hADSCs

**Fig. 1:**
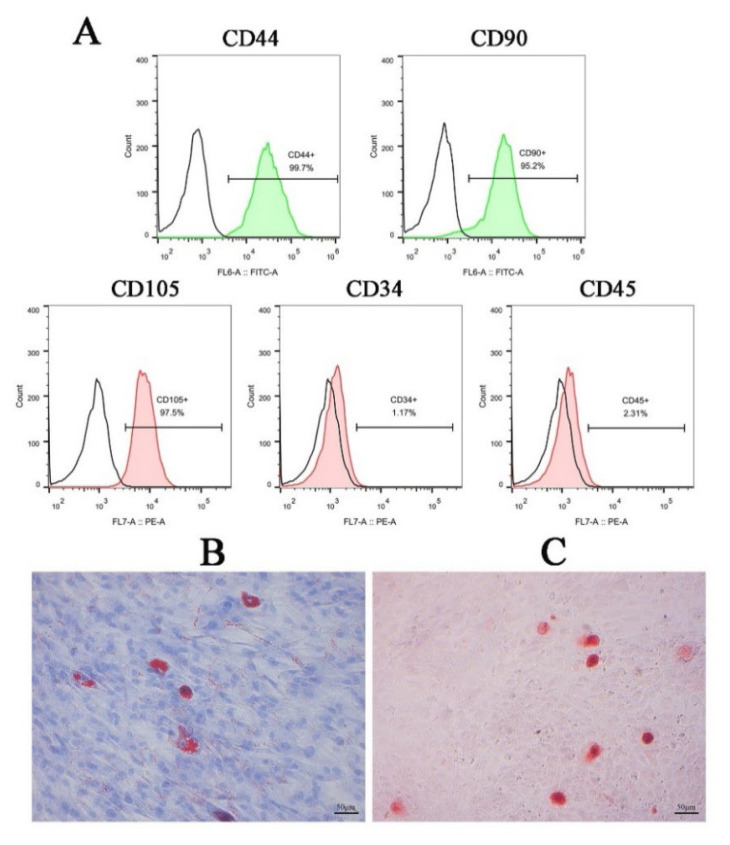
Identification of hADSCs. (**A**) Flow cytometric analysis of surface markers on hADSCs. (**B**) Oil Red O staining following adipogenic differentiation induction (Scale bar=50 μm). (**C**) Alizarin Red staining following osteogenic differentiation induction (Scale bar=50 μm).

To enhance the scientific rigor of our conclusion, we isolated hADSCs from human adipose tissue using enzymatic digestion. Subsequently, we characterized their surface markers *via* flow cytometry and confirmed their multidirectional differentiation potential through adipogenic and osteogenic induction assays. As shown in Figure 1, flow cytometry analysis revealed that the hADSCs expressed high levels of mesenchymal stem cell markers CD44 (99.27±2.38 %), CD90 (94.83±4.86 %), and CD105 (97.83±4.21 %). Conversely, hematopoietic stem cell markers CD34 (1.18±0.23 %) and CD45 (2.32±0.30 %) were minimally expressed (Fig. 1A). Additionally, staining with Oil Red O and Alizarin Red demonstrated the presence of orange lipid droplets (Fig. 1B) and calcium deposition (Fig. 1C) following adipogenic and osteogenic induction, respectively. These findings collectively confirm that the isolated cells possess characteristic features typical of stem cell type.

### Effects of different wavelengths of light on the proliferation and secretion of angiogenic factors of hADSCs

**Fig. 2:**
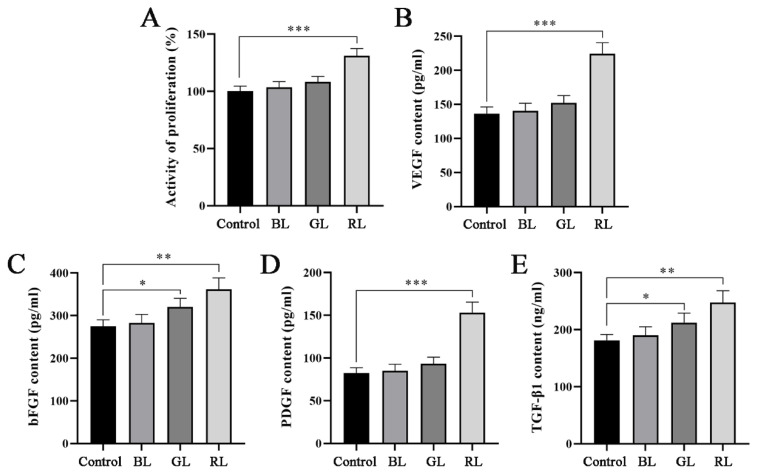
Effects of different wavelengths of light on the proliferation and secretion of angiogenic factors of hADSCs. (**A**) CCK-8 was used to detect the proliferation activity of cells in each group. (**B–E**) ELISA was used to detect the contents of VEGF, bFGF, PDGF and TGF-β1 in the cell supernatant of each group. * *P*<0.05, ** *P*<0.01, *** *P*<0.001.

We irradiated hADSCs at wavelengths of 475 nm (blue light, BL), 516 nm (green light, GL), and 635 nm (red light, RL). Subsequently, we employed CCK-8 assays and ELISA to evaluate the effects of these different wavelengths on hADSCs proliferation and the secretion of angiogenic factors. As illustrated in Figure 2A, both BL and GL irradiation promoted hADSCs proliferation to a certain extent, although no significant differences were observed. In contrast, red light irradiation markedly enhanced cell proliferation activity compared to the control group. VEGF is a key mediator known to promote vascular endothelial cell proliferation, migration, and angiogenesis. bFGF, PDGF, and TGF-β1 stimulate the division and proliferation of vascular smooth muscle cells, fibroblasts, and other cell types, playing crucial roles in wound healing [19,20]. As shown in Figure 2B–2E, RL irradiation could significantly enhance the secretion of VEGF, bFGF, PDGF, and TGF-β1 in hADSCs. Therefore, we selected red light irradiation for subsequent experiments to further investigate the effects and mechanisms of light-preconditioned hADSCs on HUVECs.

### Effects of light-pretreated hADSCs on the migration and angiogenesis of HUVECs

**Fig. 3:**
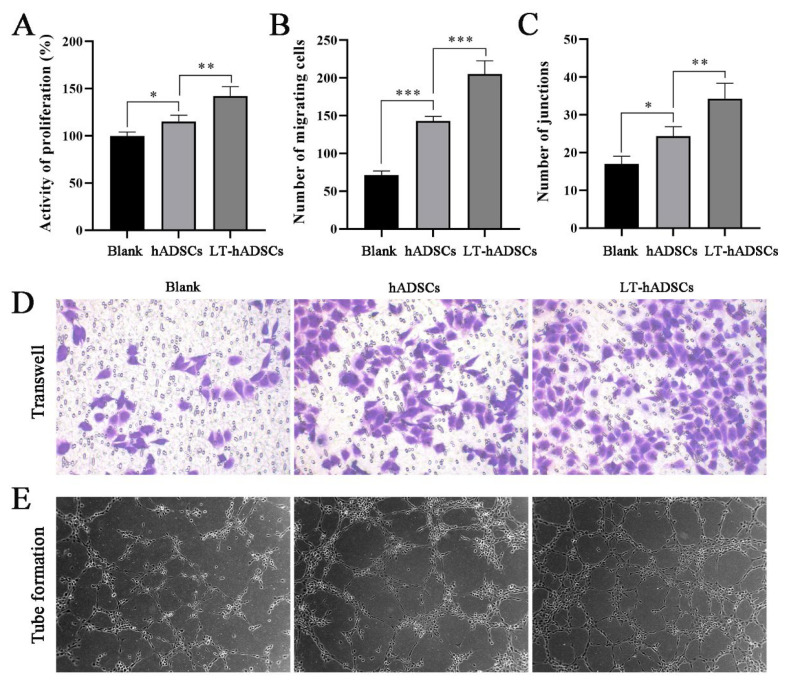
Effects of light-pretreated hADSCs on the migration and angiogenesis of HUVECs. (**A**) CCK-8 was used to detect the proliferation activity of cells in each group. (**B** and **D**). Transwell assay was used to detect the migration ability of cells in each group (×100). (**C** and **E**). Tube formation assay was used to detect the angiogenic ability of cells in each group (×40). * *P*<0.05, ** *P*<0.01, *** *P*<0.001.

Promoting the proliferation, migration, and angiogenesis of vascular endothelial cells is crucial for enhancing the success rate of parathyroid transplantation. To investigate the potential effects of light-pretreated hADSCs on parathyroid autotransplantation, we conducted *in vitro* co-culture experiments with light-pretreated hADSCs and HUVECs. We subsequently evaluated the impact of light-pretreated hADSCs on HUVECs proliferation, migration, and angiogenesis using CCK-8 assays, Transwell migration assays, and tube formation assays. As shown in Figure 3A, untreated hADSCs exhibit a promoting effect on HUVECs proliferation; however, light-pretreated hADSCs demonstrate a significantly greater stimulatory effect. Furthermore, Figure 3B to 3E illustrates that co-culturing light-pretreated hADSCs with HUVECs results in a markedly higher number of HUVECs penetrating the membrane and forming tubular branch nodes compared to co-cultures with untreated hADSCs. These findings suggest that light-pretreated hADSCs effectively enhance HUVECs proliferation, migration, and angiogenesis.

### Effects of light at different wavelengths on the expression of differentiation-related transcription factors in hADSCs

**Fig. 4:**
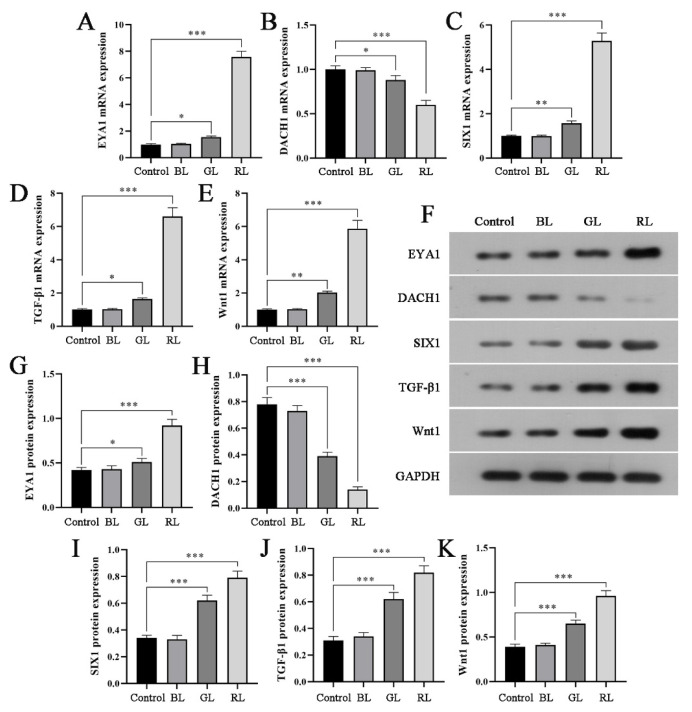
Effects of light at different wavelengths on the expression of differentiation-related transcription factors in hADSCs. (**A–E**) qRT-PCR was used to detect the mRNA expression levels of EYA1, DACH1, SIX1, TGF-β1, and Wnt1 in each group. (**F**) Protein electrophoresis pattern. (**G–K**) Western blot was used to detect the protein expression levels of EYA1, DACH1, SIX1, TGF-β1 and Wnt1 in each group. * *P*<0.05, ** *P*<0.01, *** *P*<0.001.

As adult stem cells, hADSCs not only promote angiogenesis but also induce differentiation into the affected region under specific conditions. This process involves multiple differentiation-related transcription factors. To further investigate the mechanism by which light-pretreated hADSCs enhance HUVECs migration and angiogenesis, we employed qRT-PCR and Western blot analysis to examine the expression of differentiation-related transcription factors in hADSCs exposed to different wavelengths of light. Figure 4A–4K demonstrates that RL irradiation resulted in significantly higher mRNA and protein expressions of EYA1, SIX1, TGF-β1, and Wnt1 compared to the control group, while DACH1 mRNA and protein expressions were markedly lower. RL irradiation may enhance hADSCs proliferation and the secretion of angiogenic factors by modulating differentiation-related transcription factors.

### Silencing EYA1 inhibited the secretion of angiogenic factors in hADSCs under light stimulation

**Fig. 5:**
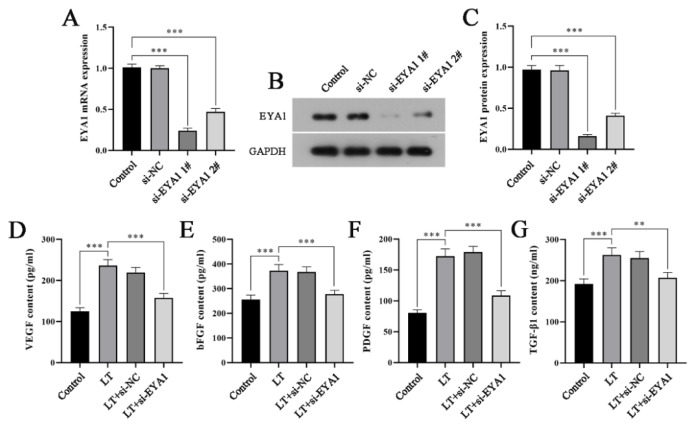
Silencing EYA1 inhibited the secretion of angiogenic factors in hADSCs under light stimulation. (**A**) qRT-PCR was used to detect the mRNA expression level of EYA1 in each group. (**B**) Protein electrophoresis pattern. (**C**) Western blot was used to detect the expression level of EYA1 protein in each group. (**D–G**) ELISA was used to detect the contents of VEGF, bFGF, PDGF and TGF-β1 in the cell supernatant of each group. ** *P*<0.01, *** *P*<0.001.

Previous studies have demonstrated that modulating EYA1 expression can enhance angiogenesis both *in vitro* and *in vivo*, thereby improving the survival rate of parathyroid transplants [12]. In conjunction with our observation of increased EYA1 expression following light treatment of hADSCs at various wavelengths, we hypothesized that EYA1 activation might represent a critical molecular mechanism underlying the promotion of HUVECs migration and angiogenesis by light-pretreated hADSCs. To test this hypothesis, we designed and synthesized EYA1-specific interference plasmids and transfected them into hADSCs. The transfection efficiency was validated using qRT-PCR and Western blot analysis. Both mRNA and protein levels of EYA1 were significantly reduced in the si-EYA1 1# and si-EYA1 2# groups compared to the control group, confirming successful construction of the EYA1 interference plasmid. Subsequently, we selected the si-EYA1 1# plasmid with superior interference efficacy for further experiments. We then transfected hADSCs with si-EYA1 and si-NC plasmids and exposed the cells to light stimulation. By specifically downregulating the expression of EYA1 in hADSCs, we investigated the impact of EYA1 silencing on the secretion of angiogenic factors under light-induced conditions. As illustrated in Figure 5D–5G, the concentrations of VEGF, bFGF, PDGF, and TGF-β1 in the supernatant of cells in the LT+si-EYA1 group were markedly reduced compared to those in the LT group. These findings indicate that EYA1 silencing significantly inhibits the secretion of angiogenic factors from hADSCs under light stimulation.

### Silencing EYA1 inhibited the promotion effect of light-pretreated hADSCs on the migration and angiogenesis of HUVECs

**Fig. 6:**
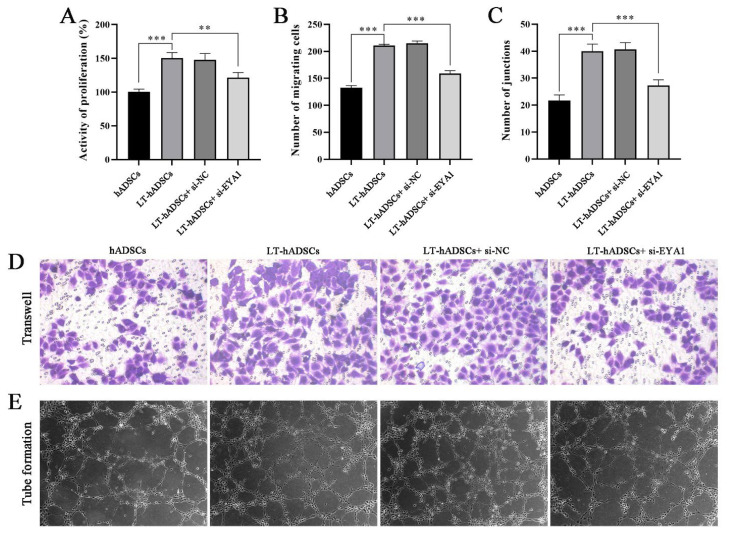
Silencing EYA1 inhibited the promotion effect of light-pretreated hADSCs on the migration and angiogenesis of HUVECs. (**A**) CCK-8 was used to detect the proliferation activity of cells in each group. (**B** and **D**) Transwell assay was used to detect the migration ability of cells in each group (×100). (**C** and **E**). Tube formation assay was used to detect the angiogenic ability of cells in each group (×40). ** *P*<0.01, *** *P*<0.001.

In prior research, our findings demonstrated that light-pretreated hADSCs can enhance the migration and angiogenesis of HUVECs, potentially through the activation of EYA1 and the secretion of angiogenic factors. To further validate this conclusion, it is necessary to investigate whether silencing EYA1 inhibits the migration and angiogenesis of HUVECs. Therefore, hADSCs transfected with si-EYA1 or si-NC plasmids were co-cultured with HUVECs following light treatment. Subsequently, CCK-8 assays, Transwell assays, and tube formation assays were conducted to evaluate the effects on HUVECs proliferation, migration, and angiogenesis. Figure 6A demonstrates that EYA1 silencing significantly inhibited the promotion of HUVECs proliferation by light-pretreated hADSCs. Additionally, Figure 6B–6E indicate that both the number of cells penetrating the membrane and the number of tubular branch nodes in the LT-hADSCs+si-EYA1 group were significantly lower compared to those in the LT-hADSCs group, suggesting that EYA1 silencing can attenuate the promotional effects of light-pretreated hADSCs on HUVECs migration and angiogenesis.

## Discussion

For post-thyroidectomy hypothyroidism, commonly employed replacement therapies are susceptible to eliciting a range of adverse reactions that can impact patient prognosis. Parathyroid autotransplantation as a means to preserve thyroid function has emerged as an efficacious treatment modality [21]. Angiogenesis, the process by which new blood vessels develop from pre-existing vasculature, plays a crucial role in wound healing and organ regeneration. This intricate process encompasses the degradation of the vascular basement membrane, followed by the proliferation and migration of vascular endothelial cells, culminating in the formation of new blood vessels and vascular networks [22,23]. Inducing tube formation in HUVECs is one of the most widely utilized and reliable methods for studying angiogenesis *in vitro*. The principle behind this method involves culturing HUVECs on basement membrane extracts to induce differentiation into tubular structures, thereby mimicking the *in vivo* process of angiogenesis [24]. In this study, HUVECs were employed as *in vitro* models to investigate a novel approach to promoting angiogenesis following parathyroid autotransplantation.

In recent years, stem cells have garnered significant attention as seed cells due to their unparalleled differentiation potential. Among these, hADSCs exhibit the capability to differentiate into multiple lineages, including chondrocytes, adipocytes, smooth muscle cells, and vascular endothelial cells. With their robust regenerative capacity, abundant sources, and ease of access, hADSCs represent a promising source of adult stem cells for differentiating into parathyroid cells [25]. Numerous studies have demonstrated that hADSCs play a crucial role in promoting angiogenesis during tissue and organ transplantation. For instance, Sun *et al.* isolated adipose-derived stem cell-derived extracellular vesicles (ADSC-EVs) from hADSCs and injected them into mice transplanted with human adipose tissue. The results indicated that ADSC-EVs significantly enhanced angiogenesis and fat regeneration post-transplantation [26]. Pilny *et al.* administered hADSCs into the gastrocnemius muscle of hindlimb ischemia model mice, observing that hADSCs transformed macrophages within the tissue to an M2 phenotype *via* secretion of interleukin-6, thereby mitigating inflammation and activating the angiogenic process to repair damaged muscle [27]. Additionally, Bi *et al.* created a 6-mm-diameter wound on the backs of hyperglycemic mice and monitored the wound for 9 days following subcutaneous injection of hADSCs. They found that hADSCs promoted wound healing by enhancing fibroblast migration and capillary structure formation in HUVECs [28]. These findings suggest that hADSCs may enhance the success rate of parathyroid transplantation through promotion of HUVEC migration and angiogenesis.

In this study, we successfully isolated hADSCs from adipose tissue and characterized them by their high expression of mesenchymal stem cell markers such as CD44, CD90, and CD105, and low expression of hematopoietic markers such as CD34 and CD45, which is consistent with previous reports [17,29]. Additionally, the multipotent differentiation potential of hADSCs was confirmed through adipogenic and osteogenic differentiation assays. Subsequently, hADSCs were co-cultured with HUVECs for 24 h, revealing that hADSCs can promote HUVECs proliferation, migration, and angiogenesis to a limited extent, although the effects were not statistically significant.

Phototherapy employs monochromatic light within the wavelength range of 400 to 1100 nm to irradiate soft tissues for wound treatment. This therapeutic approach offers several advantages, including minimal invasiveness, broad applicability, and the potential for repeated treatments. The selection of parameters such as wavelength, irradiation dose, and pulse structure is critical for optimizing treatment outcomes. In recent years, various wavelengths of light have been utilized to promote wound healing and tissue regeneration. For instance, an 808 nm laser has been shown to enhance the proliferation and adhesion of epidermal stem cells and hair follicle stem cells, thereby accelerating re-epithelialization through the regulation of relevant gene expression in rats with skin defects [30]. Additionally, a 660 nm continuous-wave InGaAlP diode laser has demonstrated the ability to promote the proliferation of dental pulp stem cells under nutrient-deficient conditions [31]. Furthermore, phototherapy has garnered significant attention for its potential to enhance the differentiation potential of hADSCs, thereby amplifying their role in regenerative medicine. Zare *et al.* investigated the effects of four different wavelengths on hADSCs and found that 630 nm and a combination of 630 nm + 810 nm significantly stimulated hADSCs proliferation while inhibiting apoptosis [32]. Mvula *et al.* conducted a co-culture experiment with hADSCs and smooth muscle cells at a 1:1 ratio, followed by irradiation with a 636 nm laser, which promoted the differentiation of hADSCs into smooth muscle cells [33]. Liao *et al.* reported that low-intensity laser irradiation serves as an effective biostimulant for hADSCs, enhancing their proliferation, adipogenic differentiation, and secretion of growth factors. Furthermore, light-pretreated hADSCs have been shown to significantly reduce epidermal thickness while increasing dermal thickness in photoaged mouse skin [34]. These findings suggest that light pretreatment may potentiate the stimulatory effects of hADSCs on the proliferation, migration, and angiogenesis of HUVECs.

Therefore, we employed light sources with wavelengths of 475 nm, 516 nm, and 635 nm to irradiate hADSCs. The results demonstrated that red light at a wavelength of 635 nm had the most significant effect on promoting hADSCs proliferation and stimulating the secretion of angiogenic factors such as VEGF, bFGF, PDGF, and TGF-β1. Furthermore, hADSCs pretreated with light exhibited markedly enhanced effects on the proliferation, migration, and angiogenesis of HUVECs compared to untreated hADSCs. Although our findings suggest that light may promote HUVECs angiogenesis by inducing hADSCs to secrete angiogenic factors, the precise molecular mechanisms underlying this process remain unclear and warrant further investigation.

As previously discussed, EYA1 may play a role in the process by which hADSCs stimulate HUVECs angiogenesis. Moreover, our observations indicate that different wavelengths of light influence the expression of multiple differentiation transcription factors in hADSCs, with red light demonstrating the most significant effect. Specifically, exposure to light resulted in increased mRNA and protein expression levels of EYA1, SIX1, TGF-β1, and Wnt1, while the mRNA and protein expression of DACH1 was reduced. EYA1, as part of the EYA1/SIX1/DACH1 genetic network, coactivates with SIX1 in the nucleus to promote parathyroid tissue development [35] and functions as a novel co-regulator of TGF-β1 during epithelial-mesenchymal transition, regulating cell proliferation and tissue differentiation [36]. Additionally, DACH1 is involved in artery and capillary formation [37]. However, current studies on the synergistic effects of EYA1, SIX1, and DACH1 in promoting angiogenesis predominantly focus on cancer-related aspects [38,39], leaving the role of these genes in normal tissue differentiation incompletely understood. Both TGF-β1 and Wnt1 are pivotal regulators in inducing cell differentiation. Activation of their respective signaling pathways can enhance the proliferation of HUVECs and stimulate the secretion of various angiogenic factors, including VEGF [40,41], findings that align with our experimental results. To investigate the role of EYA1 in light-pretreated hADSCs promoting HUVECs migration and angiogenesis, we transfected an EYA1-specific interference plasmid into hADSCs and subsequently co-cultured them with HUVECs. The results demonstrated that silencing EYA1 significantly attenuated the pro-migratory and pro-angiogenic effects of light-pretreated hADSCs on HUVECs. These findings suggest that light-induced activation of hADSCs may promote the expression of growth and differentiation factors such as TGF-β1 and Wnt1 by upregulating EYA1 transcription, thereby enhancing the secretion of angiogenic factors and stimulating HUVECs migration and angiogenesis.

## Conclusions

In this study, we demonstrated that light-pretreated hADSCs enhanced the migration and angiogenesis of HUVECs, likely through the activation of EYA1 which promotes the secretion of angiogenic factors. This finding offers a novel strategy to improve the survival rate of parathyroid transplants. However, this study has certain limitations. While our focus was on HUVECs, wound healing and tissue growth involve multiple cell types, including fibroblasts and epidermal stem cells. Future research will aim to comprehensively validate these findings across a broader range of cellular models.
